# Supplementary material for: Determinant of zinc deficiency in orthopaedic inpatients
Source: PLoS One. 2025 May 9;20(5):e0322142. doi: 10.1371/journal.pone.0322142 (PMC12064024; doi:10.1371/journal.pone.0322142)
Supplement: Attachment — Submitted filename: Comment to the author.docx [file pone.0322142.s011.docx]

**Comments to the author**

Hip fracture is a predictive factor for zinc deficiency in orthopedic inpatients.

**Title:**

- I suggest changing the title to “Determinant of Zinc Deficiency in Orthopedic Inpatients” for clarity.

**Abstract**

- Page 2, line 40: The study aimed to investigate the incidence of zinc deficiency. How did you assess the incidence with a case-control study design, given that you predetermined the number of case and control groups?

**Introduction**

- The introduction is well-written, but please add details on the burden, consequences, and associated factors of the problem.
- Page 3, line: Please clarify what you mean by retrospective case-control study design; it is unclear.

**Materials and Methods**

- Page 4 line 129 “demographics (gender, age, height, weight, body mass index [BMI]),” How did you measure the weight and height of the patient. Which instrument was used?
- If your focus is on incidence, which measures did you use: incidence density or cumulative incidence?
- What analysis method did you employ to estimate incidence?
- What effect measures did you use, and how was the analysis conducted?

**Results**

- While you covered much content, you overlooked an essential component—interpretation of the predictive factors of zinc deficiency.
- How did you control for confounding?
- If you performed multivariate logistic regression analysis, please provide the values and regression table that includes both adjusted and crude odds ratios.

**Discussion**

- If you used a case-control study design, why focus on the prevalence of the problem?
- The discussion requires further synthesis and should concentrate more on the predictive factors.
- What was the strength and the limitation of your study? Because it is the base for future research.
